# Supplementary material for: The novel spore-specific regulator SscA controls Aspergillus conidiogenesis
Source: mBio. 2023 Sep 14;14(5):e01840-23. doi: 10.1128/mbio.01840-23 (PMC10653911; doi:10.1128/mbio.01840-23)
Supplement: Supplemental material — Captions for supplemental tables; Figures S1 to S10. [file mbio.01840-23-s0001.docx]

**Supplemental files**

**Table S1.** The expression levels of all genes in the hyphae and conidia of *A. nidulans*.

**Table S2.** The expression levels of all genes in the hyphae and conidia of *A. flavus*.

**Table S3** The expression levels of all genes in the hyphae and conidia of *A. fumigatus*.

**Table S4**. Gene Ontology (GO) term enrichment analysis of genes differentially expressed in three *Aspergillus* conidia (Biological process).

**Table S5.** Cell wall-related genes in three *Aspergillus* species conidia.

**Table S6.** The expression levels of all genes in the conidia of *A. nidulans* wild-type and Δ*sscA* mutant.

**Table S7.** Gene Ontology (GO) term enrichment analysis of genes differentially expressed in Δ*sscA* conidia (Biological process).

**Table S8.** Differentially expressed genes in secondary metabolite genes clusters in the Δ*sscA* conidia.

**Table S9.** *Aspergillus* strains used in this study.

**Table S10.** Oligonucleotides used in this study.

**Figure S1. The transcriptional profiles of conidia are different from those of hyphae in three representative *Aspergillus* species.** Heatmap plot showing differential expression genes between hyphae and conidia of *A. nidulans*, *A. flavus*, and *A. fumigatus*.

**Figure S2. The analysis of the transcription factors in three *Aspergillus* species.**  **(A~C)** Donuts charts showing the number of total transcription factors (TFs) encoded by *A. nidulans* (A), *A. flavus* (B), and *A. fumigatus* (C).

**Figure S3. The mRNA expression levels of twenty-four spore-specific transcription factors (TFs) during the life cycle of the *A. nidulans*.** The transcript levels of each TF were measured during vegetative growth (V) and post-asexual developmental induction (A).

**Figure S4. Point-phenotypes of twenty-four transcription factors (TFs) deletion mutant strains.** The putative spore-specific TF-disrupted mutants were generated at least three independent strains using the homologous recombination method. Those were point-inoculated on solid MM at 37 °C for 5 days in light conditions.

**Figure S5. The analysis of syntenic genes around *sscA* gene.** The syntenic genes around *sscA* genes in three *Aspergilli* genome. The expression level of each gene was presented as log2 fold change (conidia/hyphae) in each *Aspergillus* species (Scale bar = 0.25 kilobase pair). The dotted line represents the homologous genes in three *Aspergillus* species. Dash indicates that the corresponding gene was not significantly expressed between conidia and hyphae of each strain.

**Figure S6. Effects of SscA on mycelia. and conidial color.**

**(A)** Color of the growth medium of wild-type (WT), Δ*sscA*, and complementary (Cʹ *sscA*) strains grown on solid minimal media (MM) at 37°C for 5 days. **(B)** Conidial color extracted from wild-type (WT), Δ*sscA*, and complementary (Cʹ *sscA*) strains. **(C-D)** Quantitative real-time RT-PCR analysis of *yA* (C) or *wA* (D) gene in WT, Δ*sscA*, C’ *sscA* strains after inducing asexual development and conidia. The mRNA expression was normalized to that of the endogenous control β-actin gene. The control group was expression levels of wild-type 12 hour after inducing asexual development (****p*<0.001, ***p*<0.01, **p*<0.05).

**Figure S7. Transcriptome analyses of the differential genes in Δ*sscA* conidia.** **(A)** Heatmap plot presenting differential expressed genes (DEGs) between WT and Δ*sscA* conidia (|Log2FC|≥1 and p-value <0.05). **(B)** The expression patterns of genes around the *sscA* genes in *A. nidulans* genome. The expression level of each gene was presented as log_2_ fold change (conidia/hyphae) in WT and Δ*sscA* conidia (Scale bar = 1 kilo base pair).

**Figure S8. Effects of SscA on cell wall integrity. (A)** Fungal strains were point-inoculated on MM with caspofungin and cultured at 37 °C for 5 days. **(B)** Quantitative analysis of stress sensitivity shown in (A). The stress sensitivity was calculated the ratio of fungal growth of treated relative to that of untreated control (**p*<0.05). **(C-E)** Heatmap diagram showing relative transcript abundance of genes involved in chitin (C), hydrophobins (D) or melanin (E) in *sscA* deletion strain as compared with wild-type strain.

**Figure S9. The analysis of sterigmatocystin production in Δ*sscA* mycelia.** **(A)** Thin layer chromatography (TLC) image of ST extracted from WT, Δ*sscA*, C’ *sscA* mycelia. Each strain was inoculated into 5 mL of liquid complete medium (CM) and incubated at 30 °C for 7 days. **(B)** Bar plot showed the relative intensity of the ST bands analyzed in (A) (**p*<0.05).

**Figure S10. The expression patterns of secondary metabolites biosynthetic genes in Δ*sscA* conidia.** The heatmap showing transcript levels of asperthecin **(A)**, emericellamide **(B)**, and terrequinone **(C)** gene clusters in Δ*sscA* conidia.

**Figure S11. The genetic relationship between AbaA and SscA. (A)** Phenotypes of WT and Δ*abaA* strains following the induction of asexual development. **(B)** Quantitative real-time RT-PCR analysis of *sscA* gene in WT and Δ*abaA* strains after inducing asexual development. The mRNA expression was normalized to that of the endogenous control β-actin gene (****p*<0.001).

**Figure S12. Construction of *sscA-*overexpressed strain.** **(A)** Verification of *sscA*-overexpressed strains (OE*sscA*). Control and OE*sscA* candidates were cultured on liquid MMG (non-inducing) and MMT (inducing) and verified using quantitative RT-PCR (qRT-PCR). The fold expression was computed using 2^-ΔΔCt^ method (****p*<0.001). **(B)** The morphology of control and OE*sscA*. Each strain was point-inoculated on solid non-inducing and inducing media and incubated at 37 °C for 5 days.

**
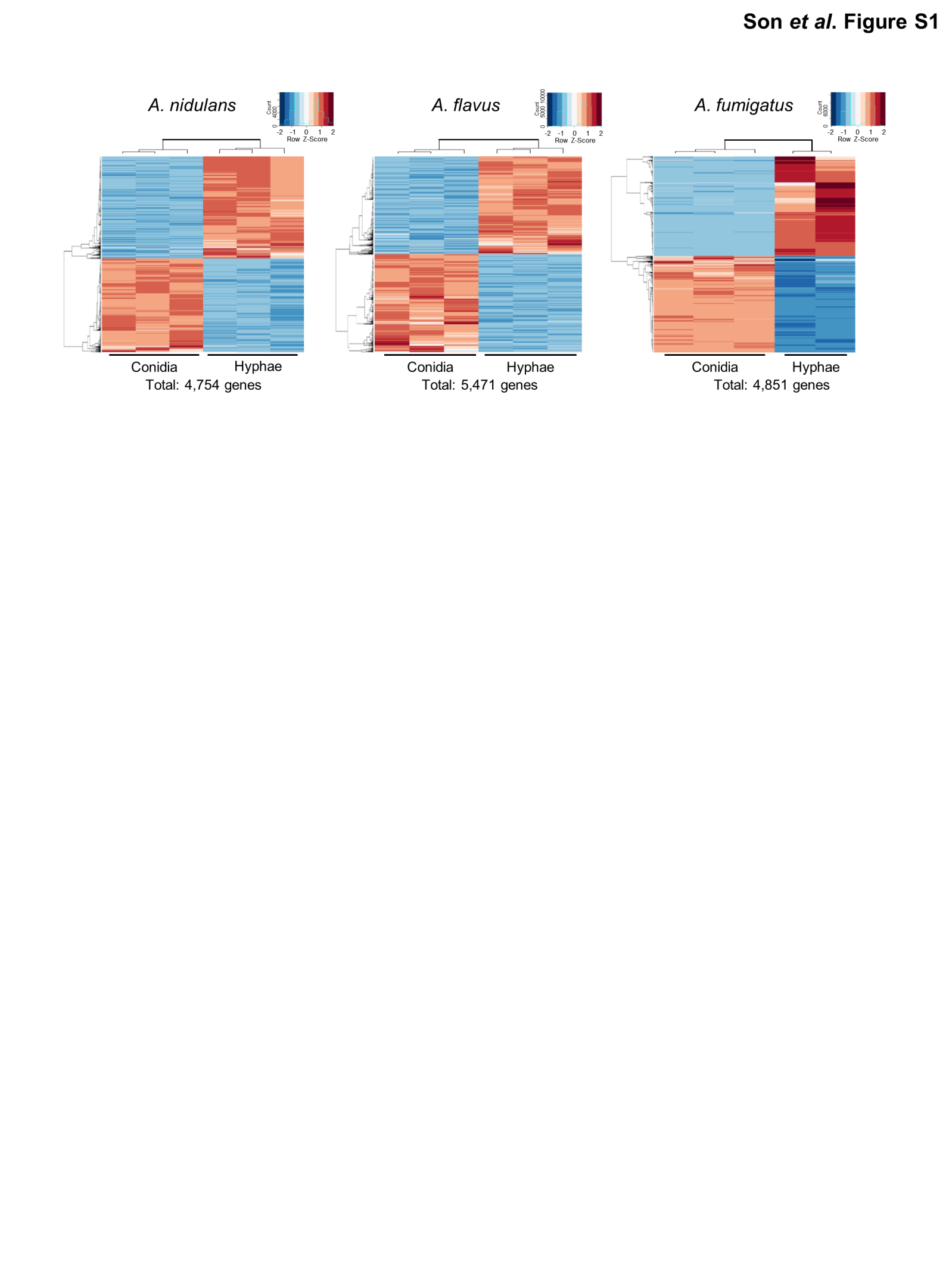

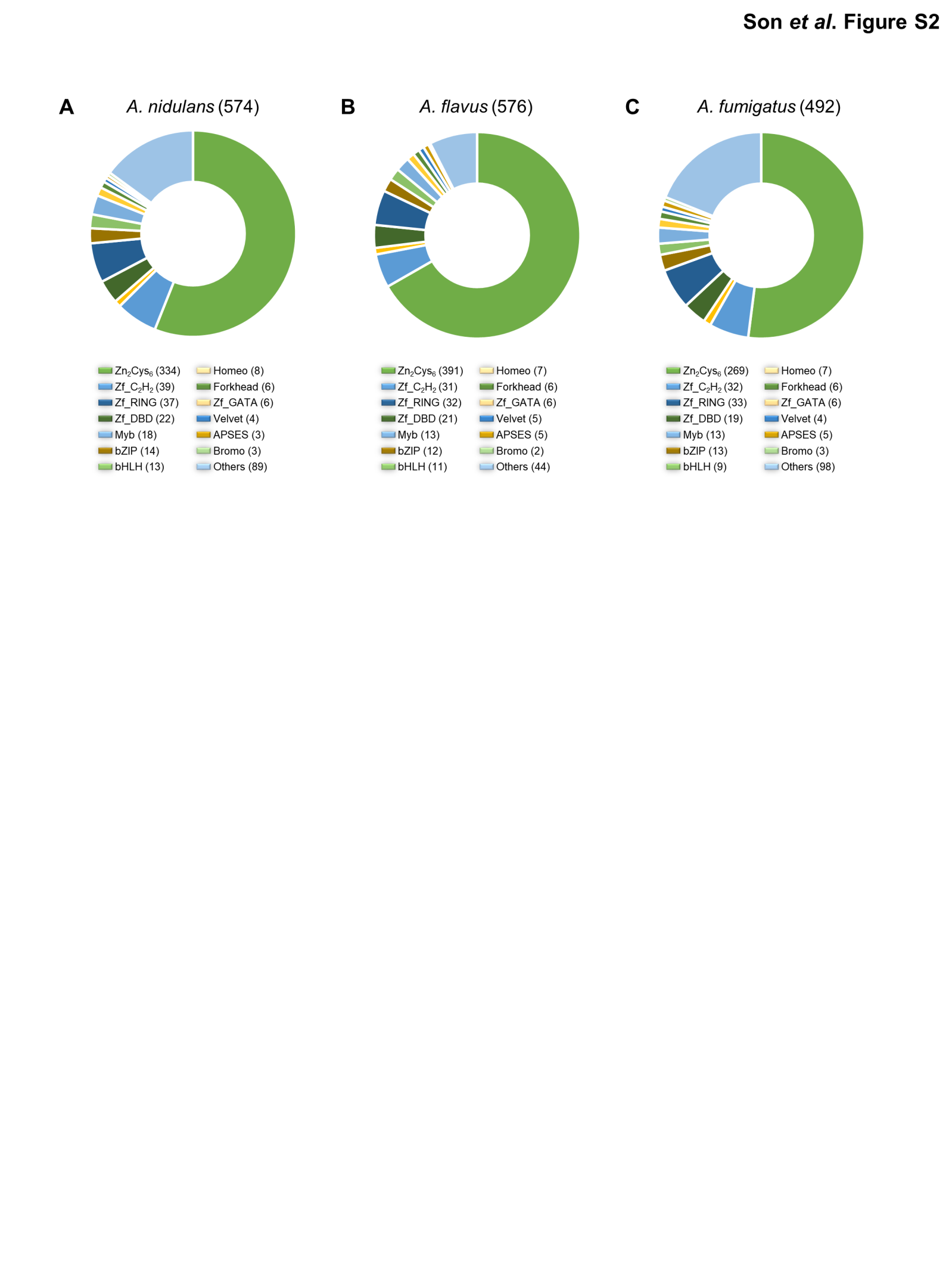

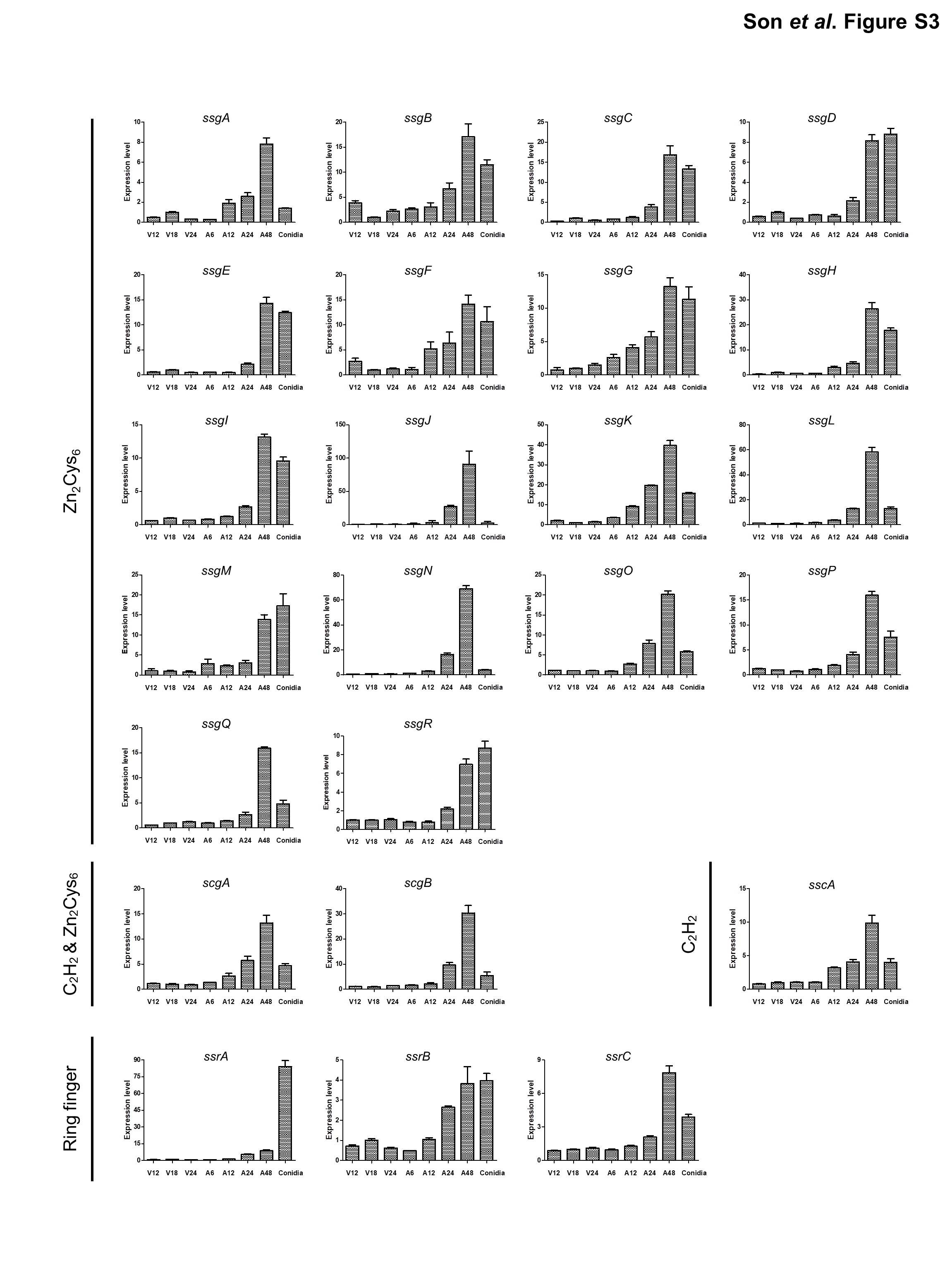

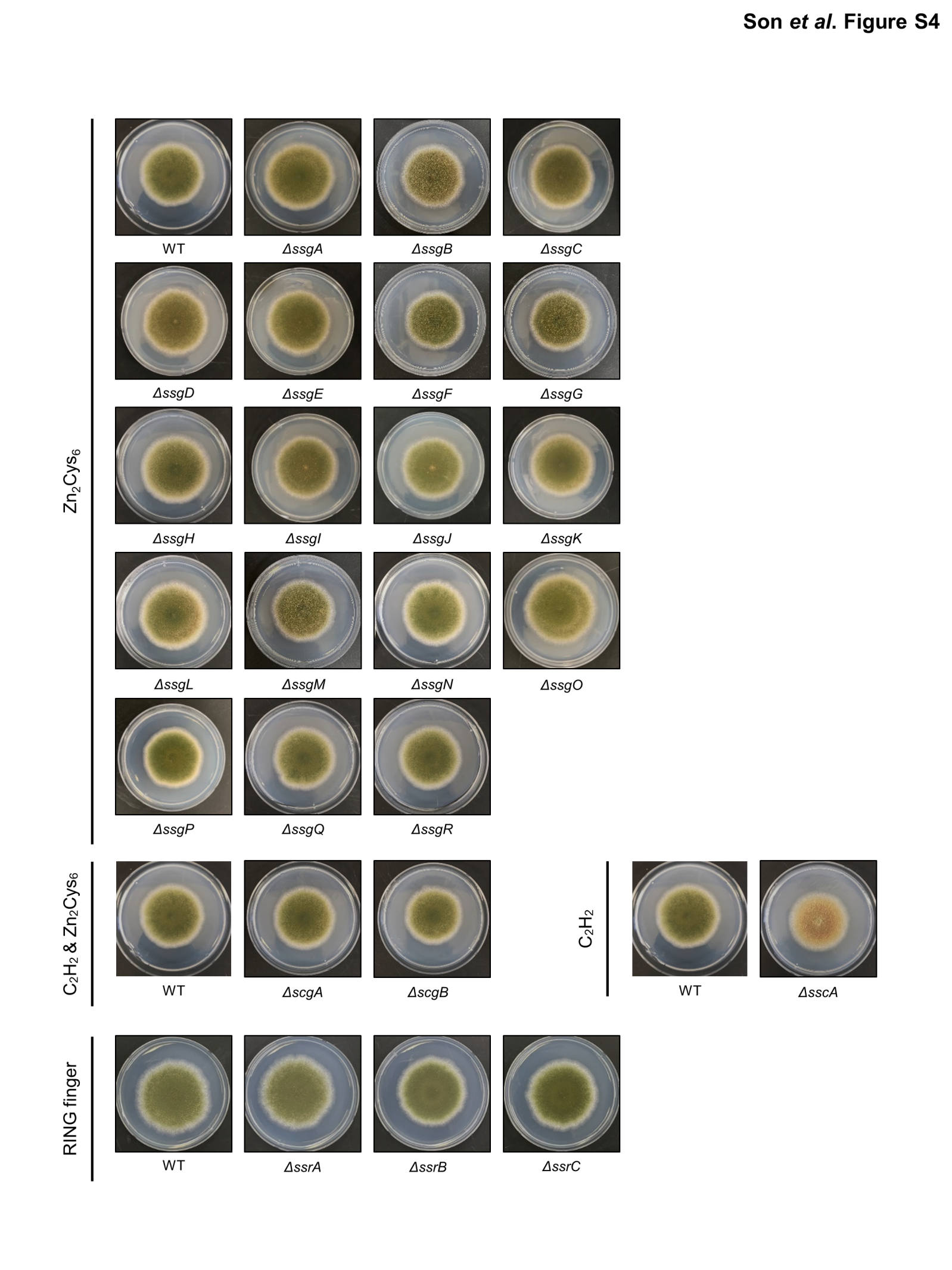

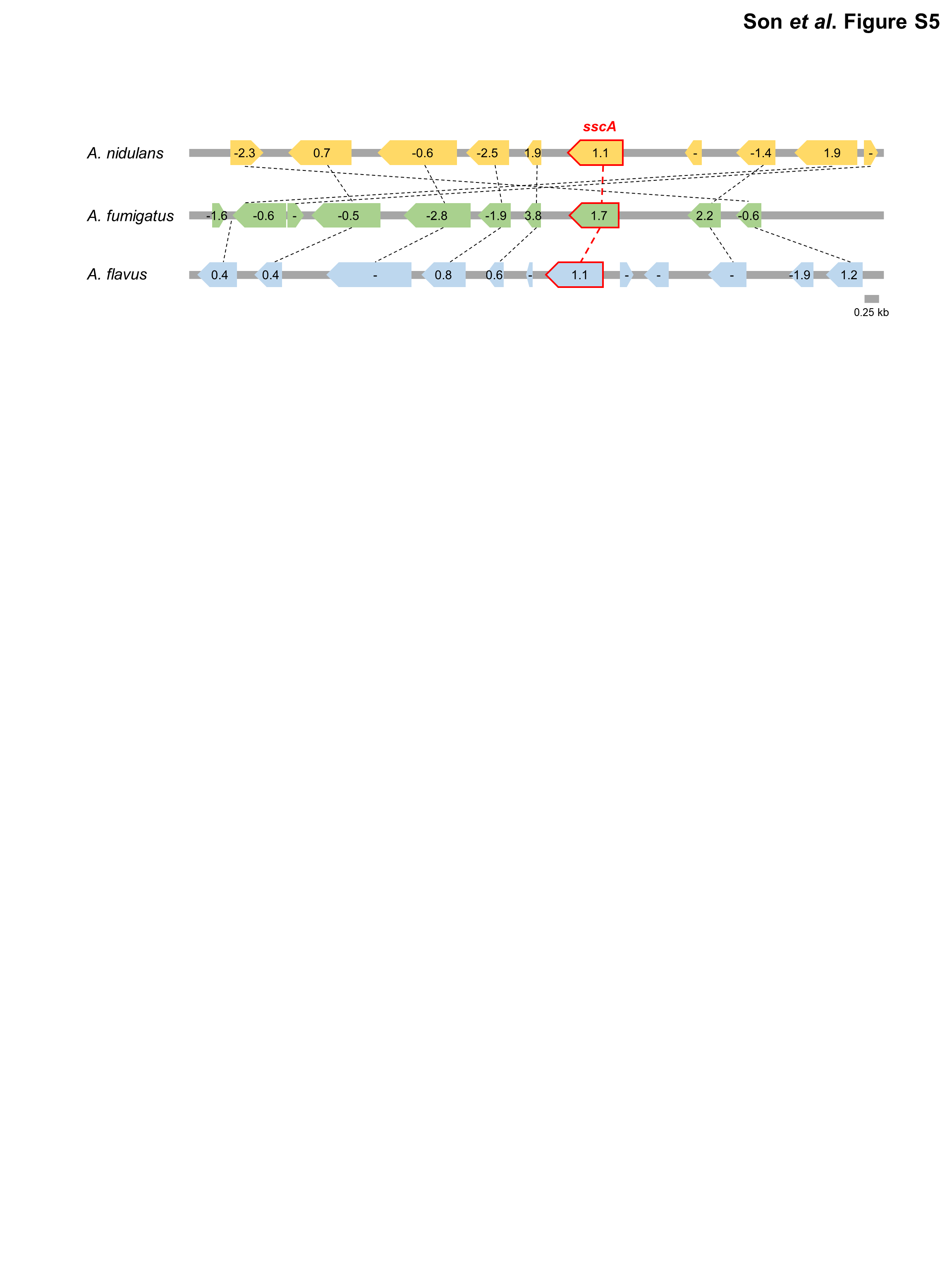
**

**
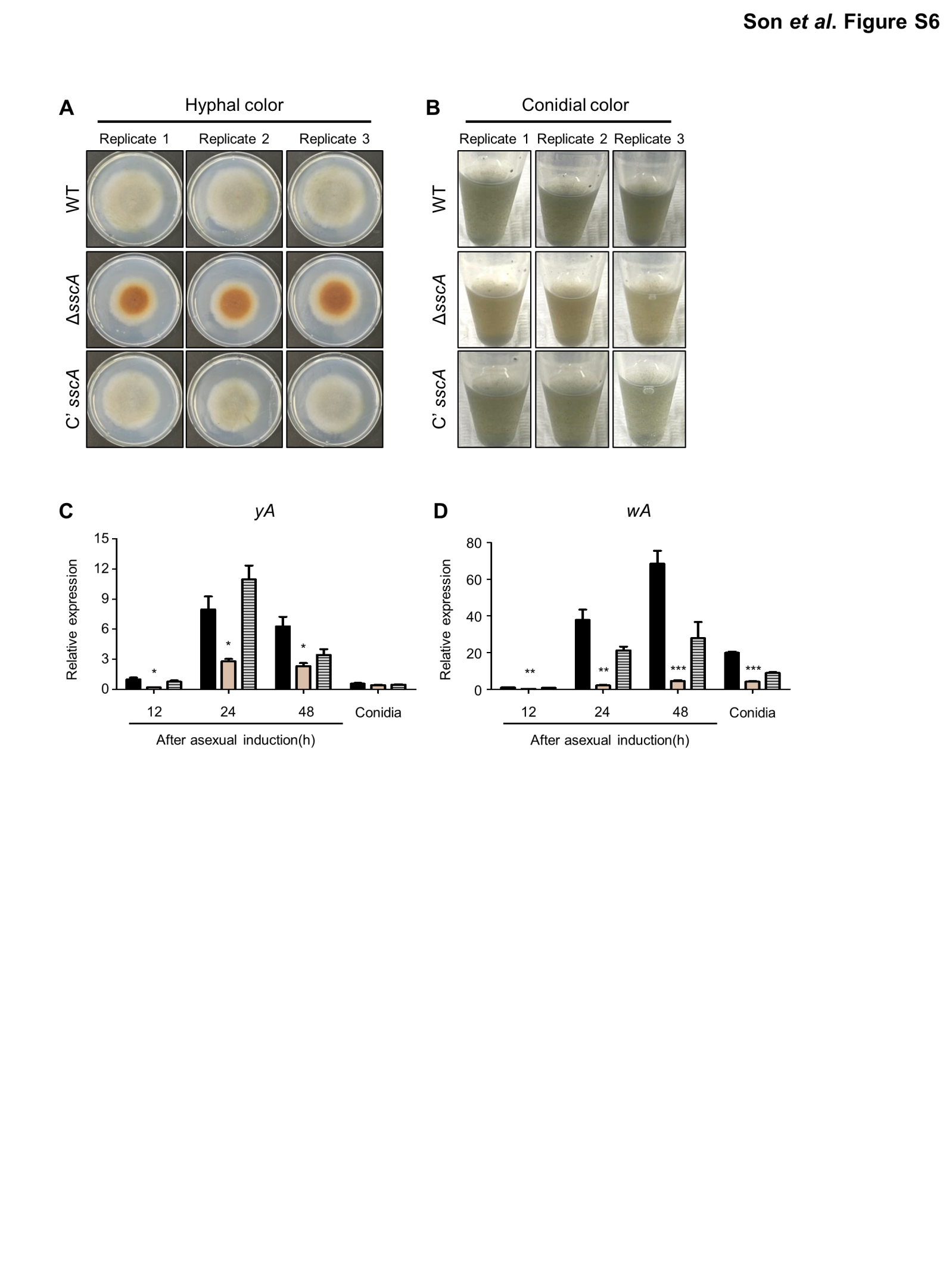
**

**
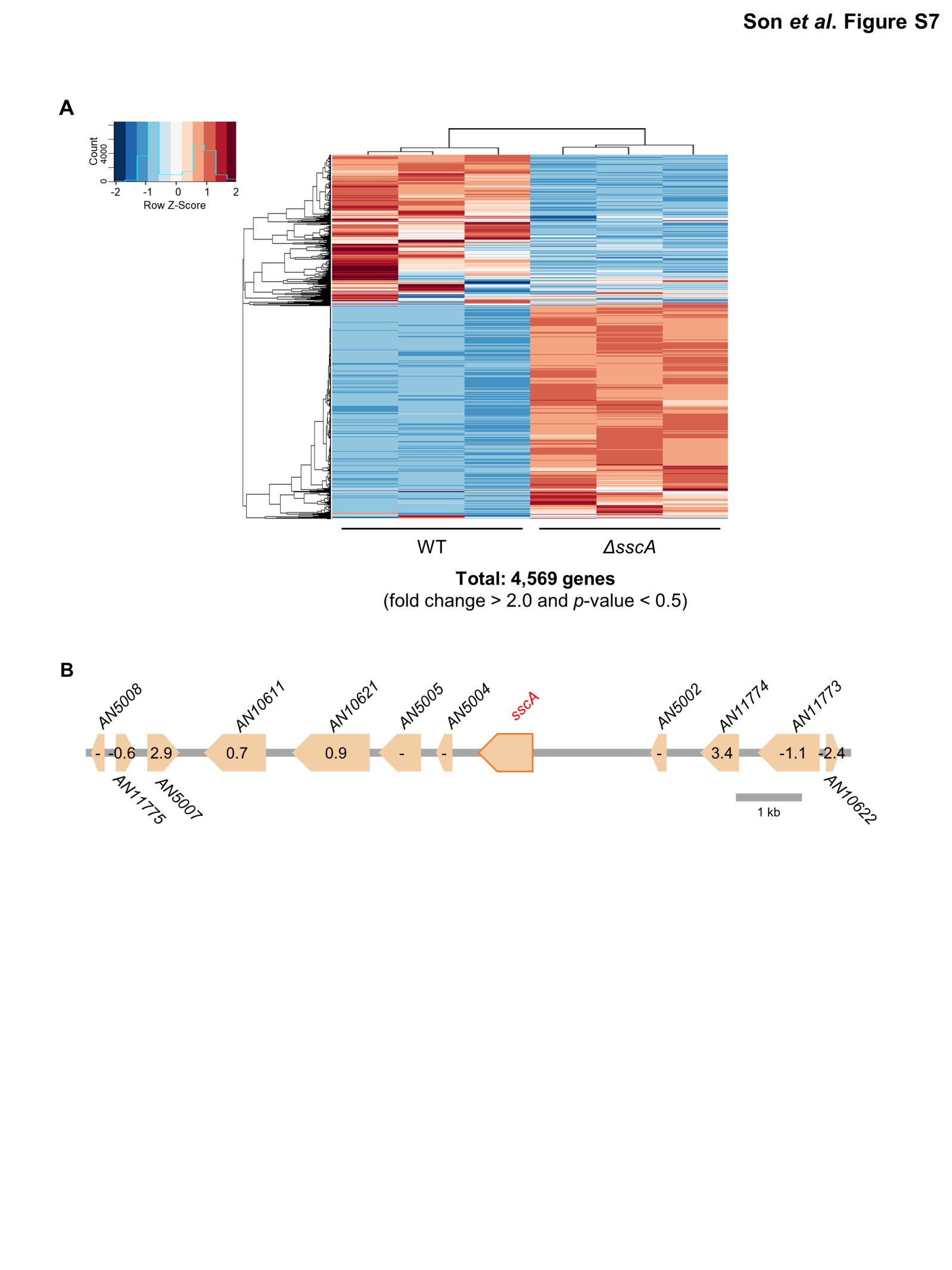

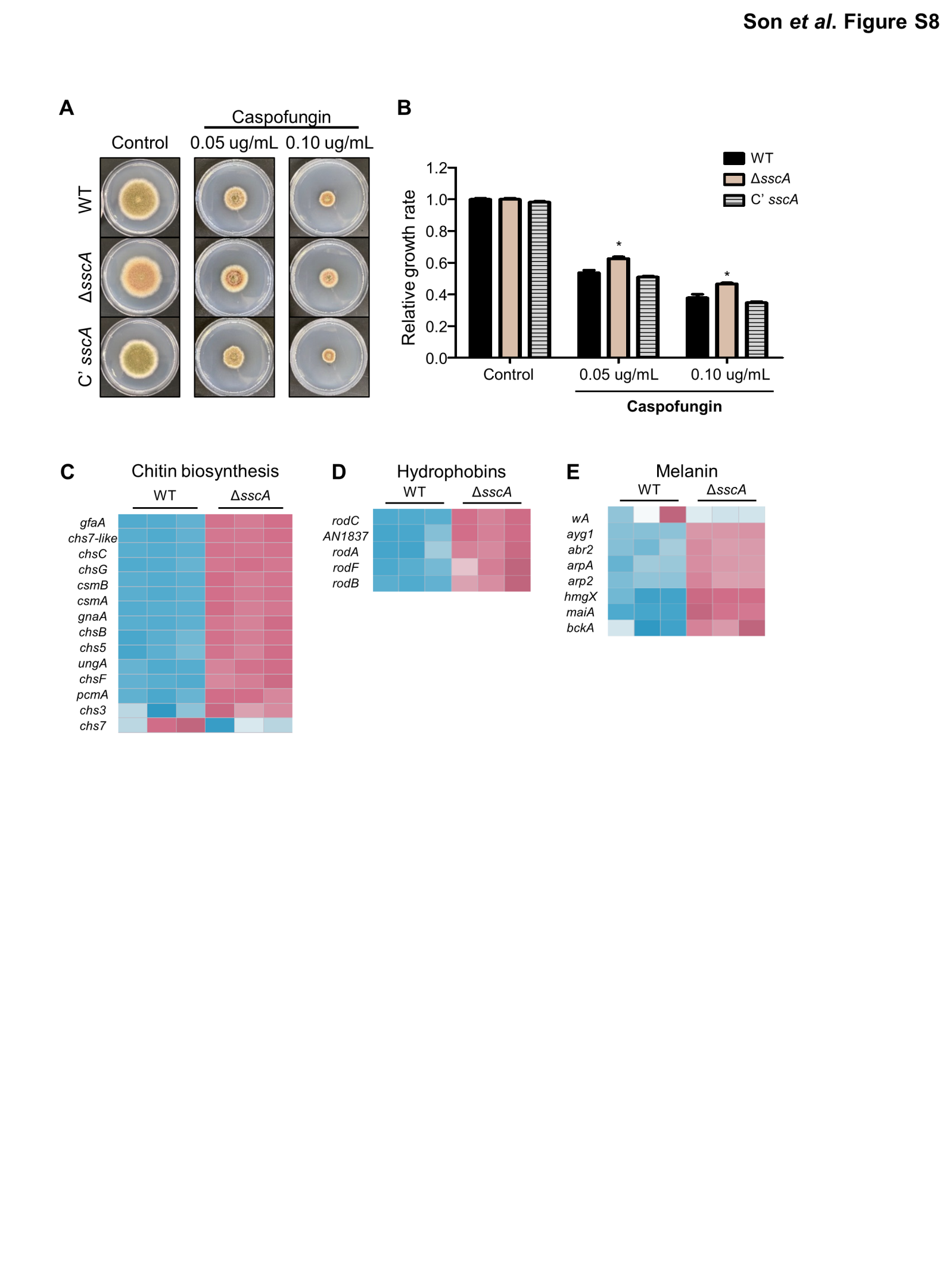

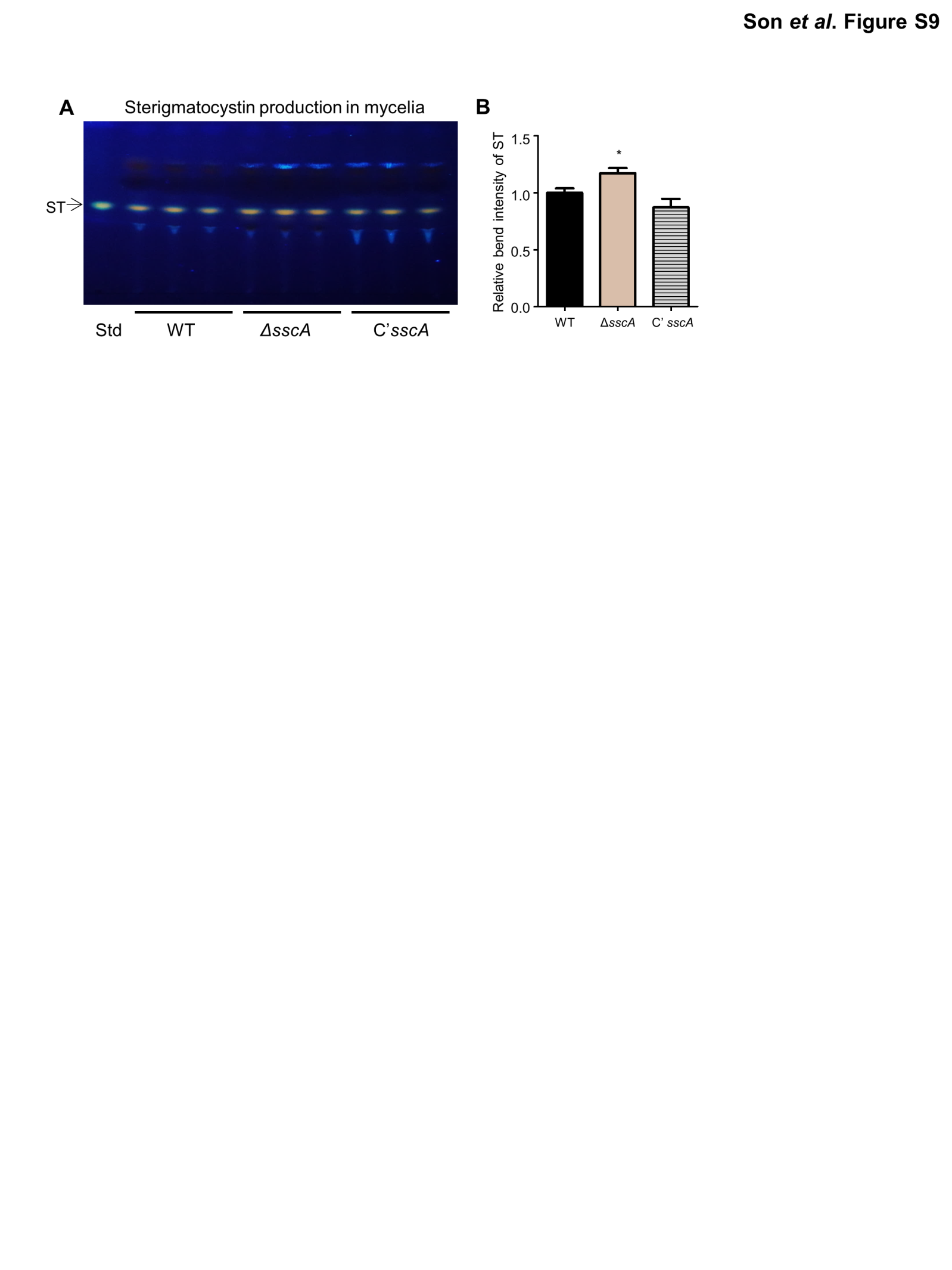

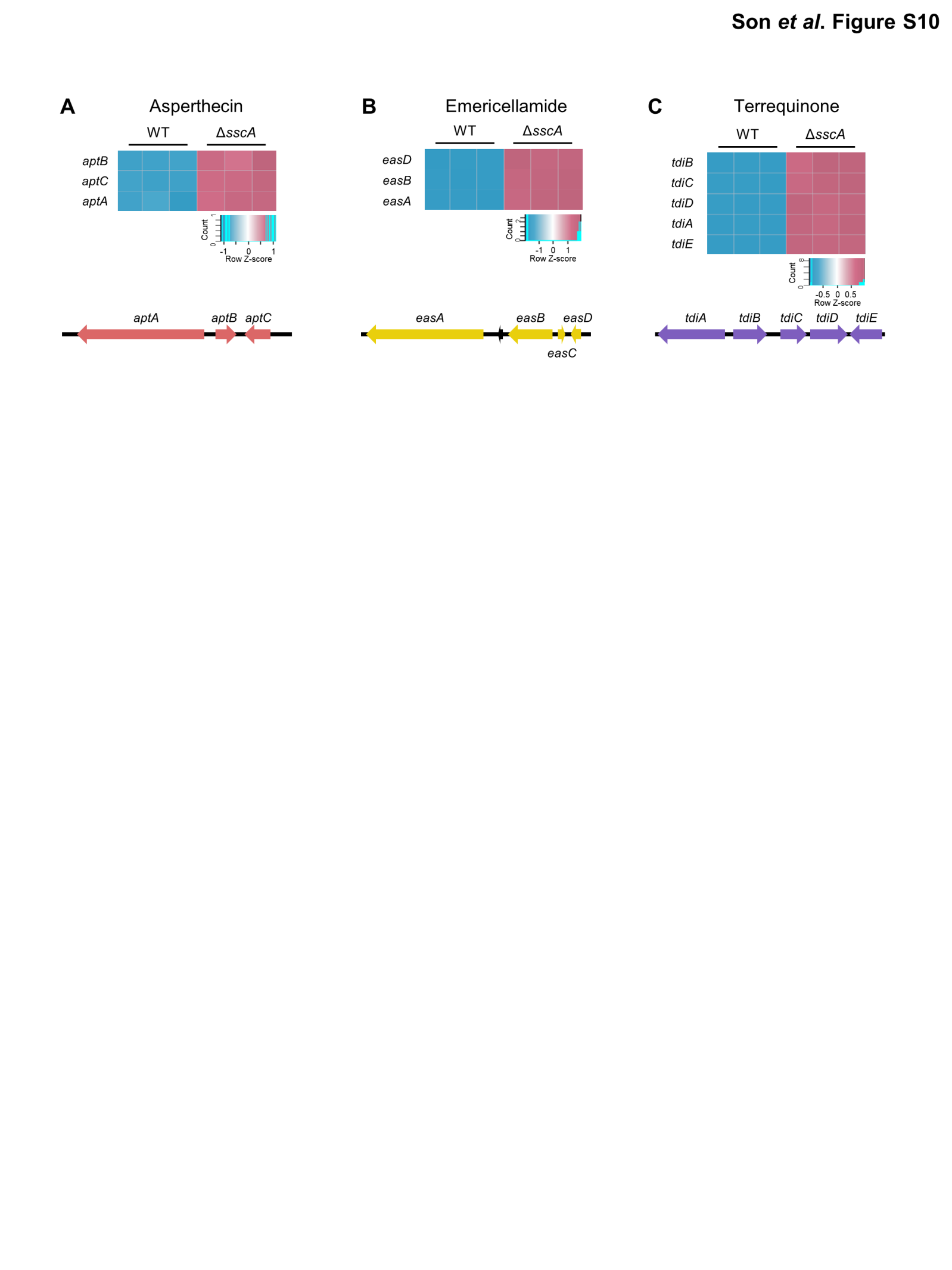

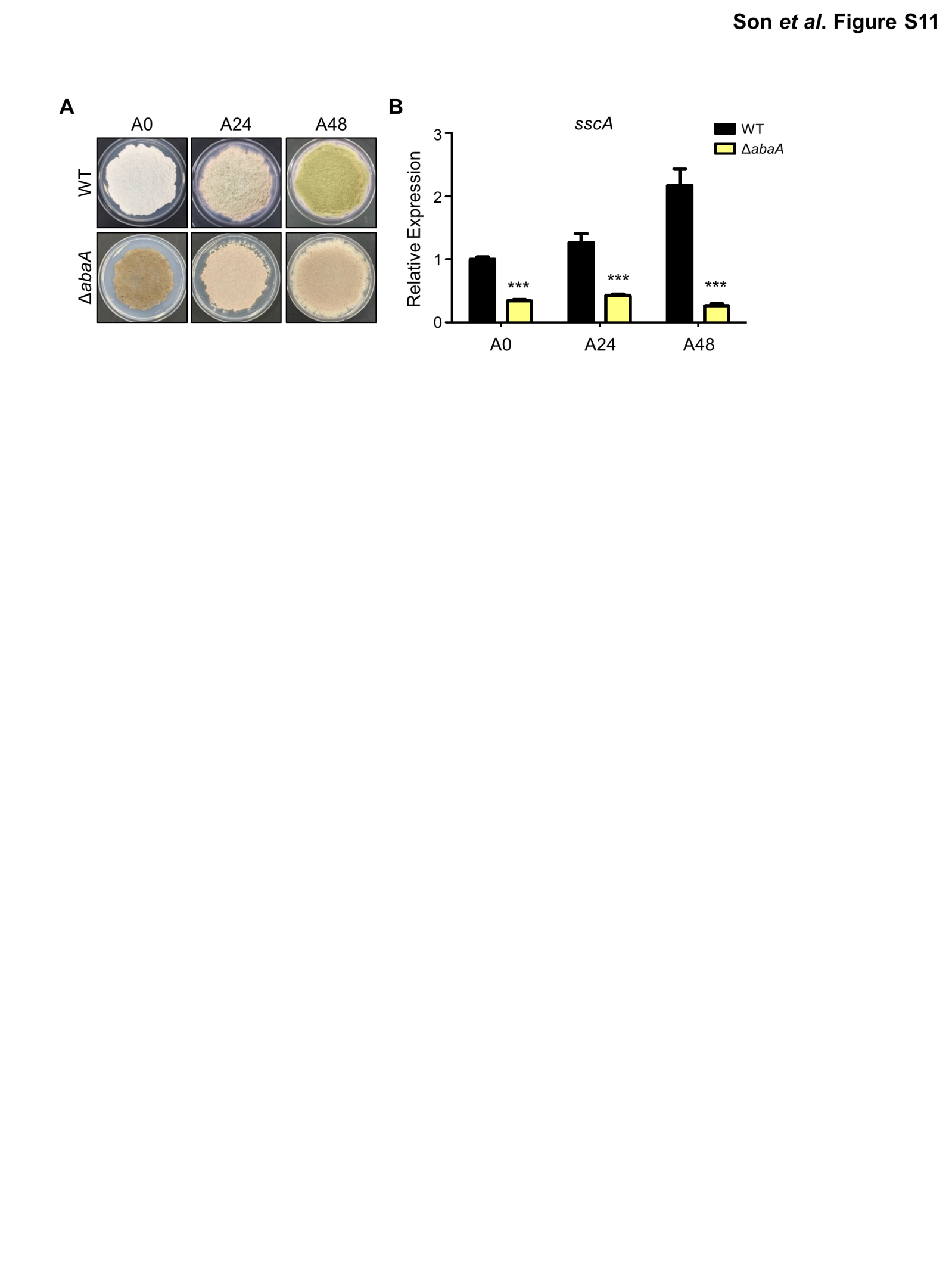

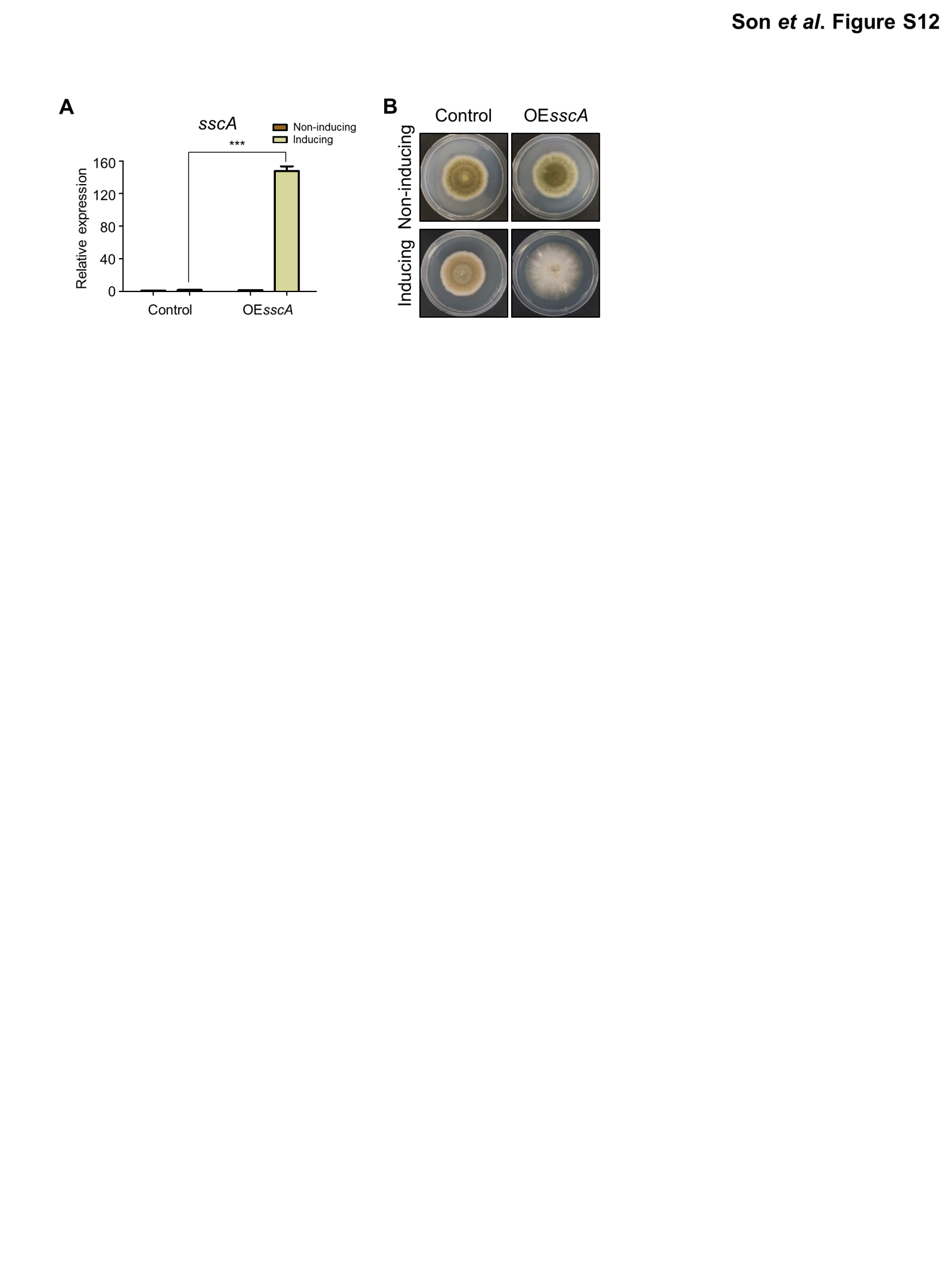
**
